# Remimazolam anaphylaxis in a patient not allergic to brotizolam: a case report and literature review

**DOI:** 10.1186/s12871-024-02591-w

**Published:** 2024-06-08

**Authors:** Toshihiro Nakai, Eisuke Kako, Haruko Ota, MinHye So, Kazuya Sobue

**Affiliations:** Department of Anesthesiology and Intensive Care Medicine, Nagoya City University Graduate School of Medical Sciences, 1 Kawasumi Mizuho-Ku 467-8601, Nagoya, Japan

**Keywords:** Remimazolam, Anaphylaxis, Benzodiazepine, Allergy, Cross-reactivity, Bronchospasm

## Abstract

**Background:**

Remimazolam is a recently developed, ultrashort-acting benzodiazepine that is used as a general anesthetic. Some cases of remimazolam anaphylaxis have been reported, but its characteristics are not fully understood. We present an interesting case report and review of the literature to better understand remimazolam anaphylaxis.

**Case presentation:**

A 75-year-old man scheduled for robot-assisted gastrectomy was administered remimazolam for the induction of general anesthesia. After intubation, low end-expiratory CO_2_, high airway pressure and concurrent circulatory collapse were observed. Bronchoscopy revealed marked tracheal and bronchial edema, which we diagnosed as anaphylaxis. The patient suffered cardiac arrest after bronchoscopy but recovered immediately with intravenous adrenaline administration and chest compressions. We performed skin prick tests for the drugs used during induction except for remimazolam, considering the high risk of systemic adverse reactions to remimazolam. We diagnosed remimazolam anaphylaxis because the skin prick test results for the other drugs used during anesthesia were negative, and these drugs could have been used without allergic reactions during the subsequent surgery. Furthermore, this patient had experienced severe anaphylactic-like reactions when he underwent cardiac surgery a year earlier, in which midazolam had been used, but it was not thought to be the allergen at that time. Based on these findings, cross-reactivity to remimazolam and midazolam was suspected. However, the patient had previously received another benzodiazepine, brotizolam, to which he was not allergic, suggesting that cross-reactivity of remimazolam may vary among benzodiazepines. In this article, we reviewed the 11 cases of remimazolam anaphylaxis that have been described in the literature.

**Conclusions:**

Remimazolam is an ultrashort-acting sedative; however, it can cause life-threatening anaphylaxis. In addition, its cross-reactivity with other benzodiazepines is not fully understood. To increase the safety of this drug, further research and more experience in its use are needed.

**Supplementary Information:**

The online version contains supplementary material available at 10.1186/s12871-024-02591-w.

## Introduction

Remimazolam is an ultrashort-acting benzodiazepine that has been used to induce general anesthesia in Japan since August 2020, in advance of the rest of the world [1]. Remimazolam was synthesized by introducing a carboxylic ester side group into midazolam, and its chemical structure is similar to that of midazolam [2]. Compared to other benzodiazepines, remimazolam has a faster onset and less accumulation after prolonged infusion due to its shorter duration of action [3]. In addition, remimazolam is less likely to cause hypotension than propofol [4] and has been shown to be effective and safe for general anesthesia induction in high-risk surgical patients [5, 6]. Recently, several case reports of anaphylaxis caused by remimazolam have been published [7–12]. However, its clinical characteristics are not fully understood, especially in terms of cross-reactivity to other benzodiazepines.

The aim of this report is to describe a case of remimazolam anaphylaxis in a patient who was not allergic to brotizolam and to review the literature on remimazolam anaphylaxis. All applicable guidelines were followed, and written consent for publication of the case report was obtained from the patient.

## Materials and methods

A review of the literature on remimazolam anaphylaxis was conducted through the Medline/PubMed and Cochrane Library databases through October 21, 2023, searching for the key words “remimazolam”, “anaphylaxis” and “allergy”. We identified 11 cases of remimazolam anaphylaxis in 6 case reports.

## Case description

A 75-year-old man (height 157 cm; body weight 63 kg) was scheduled for robot-assisted gastrectomy for gastric cancer. His medical history included hypertension, hyperlipidemia, and emphysema. In addition, he suffered sudden circulatory collapse and bronchospasm in the ICU immediately after undergoing coronary artery bypass surgery for ischemic heart disease at 74 years of age. These symptoms were considered anaphylactic reactions caused by fresh-frozen plasma transfusion, while a severe drop in blood pressure requiring administration of noradrenaline was also observed during the induction of anesthesia. Midazolam was the only drug used during both events, but it was not considered to be a cause of anaphylaxis at that point. In addition, the patient was taking brotizolam, an oral benzodiazepine, as a sleeping pill but had no other history of allergies.

**Fig. 1:**
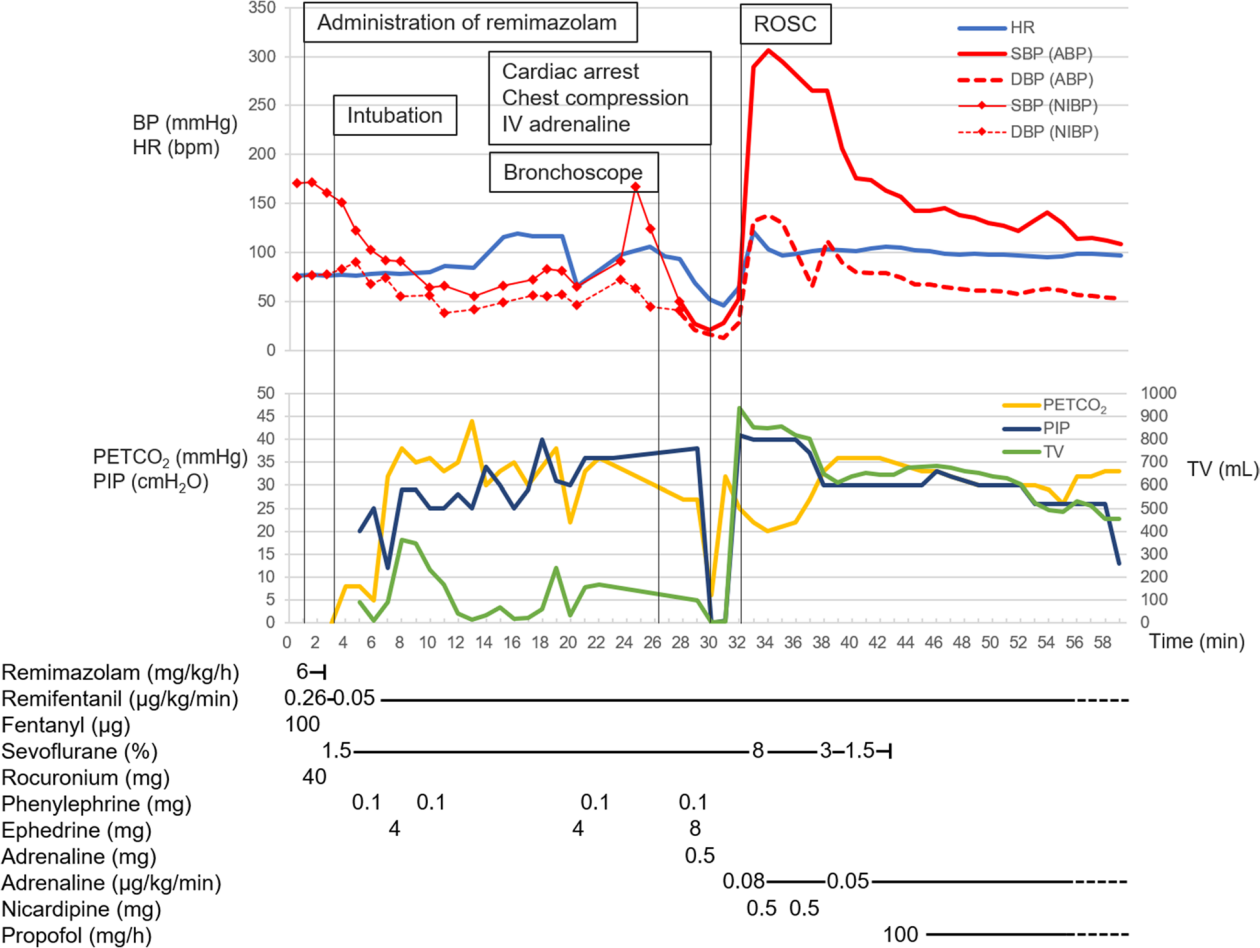
Time course of the anaphylactic episode. *BP* blood pressure, *SBP* systolic blood pressure, *DBP* diastolic blood pressure, *ABP* arterial blood pressure, *NIBP* noninvasive blood pressure, *HR* heart rate, *PETCO*_*2*_ end tidal carbon dioxide partial pressure, *PIP* peak inspiratory pressure, *TV* tidal volume*, IV* intravenous, *ROSC* return of spontaneous circulation

Before anesthetic induction, the patient’s vital signs were as follows: blood pressure, 173/73 mmHg; heart rate, 77 bpm; and SpO_2_, 98% (room air). Anesthesia was induced with intravenous fentanyl 100 mcg, remifentanil 0.26 mcg/kg/min, and remimazolam 6 mg/kg/h until loss of consciousness (total 8 mg used). Rocuronium 40 mg was administered, and he was intubated with a video laryngoscope. Tracheal intubation was successfully performed. However, the end tidal carbon dioxide was low (5–8 cmH_2_O), and more than 30 cmH_2_O of inspiratory pressure was needed to deliver the minimum necessary tidal volume (300 ml). We suspected incorrect endotracheal tube positioning, obstruction due to secretions, pneumothorax, and asthma. On auscultation, respiratory sounds in both lungs were barely audible. Endotracheal suctioning was performed, but no secretions were found. The blood pressure was also low, and inotropes and vasopressors were administered as needed (a total of 8 mg of ephedrine and 0.3 mg of phenylephrine were administered after induction of anesthesia). Bronchoscopy to check for any abnormalities in the airway revealed marked edema of the tracheal to bronchial mucosa and tracheal stenosis (Additional file 1). Based on the course and findings, we diagnosed anaphylaxis. Cardiac arrest occurred immediately after bronchoscopy; chest compressions were performed, and 0.5 mg of intravenous adrenaline was administered. Two minutes later, spontaneous circulation returned. A rash developed on the patient’s abdomen and thighs. The surgery was canceled, and he was admitted to the intensive care unit (ICU). The course of the anaphylactic episode is shown in Fig. 1. On ICU admission, the airway compliance improved, and the next day, tracheal edema was attenuated (Additional file 2), and the patient was extubated. Blood tests showed elevated tryptase levels [30 min after onset: 6.2 µg/L, 2 h after onset: 9.2 µg/L, 24 h after onset: 2.2 µg/L (normal range 1.2–5.7 µg/L)], and the acute tryptase level (2 h) was well above the patient’s baseline serum tryptase level (24 h); this finding was consistent with anaphylaxis [13]. Fentanyl, remifentanil, rocuronium and remimazolam were used just before anaphylaxis. Latex and antibiotics were not used. Four weeks later, skin prick tests for candidate drugs that might be used during the subsequent surgery were performed (Additional file 3). However, we did not perform skin prick tests for remimazolam or midazolam, which is likely to exhibit cross-reactivity with remimazolam. These drugs were strongly suspected to be the causative drugs, and the potential anaphylactic response caused by the skin test was deemed too dangerous given the cardiac function of the patient. Prick test results for all drugs were negative (Additional file 3). In addition, fentanyl, remifentanil and rocuronium had been used without anaphylactic reactions in other situations, such as during prior cardiac surgery and ICU stays (Additional file 4). Based on the above findings, we determined that the allergen was remimazolam, and intradermal testing was not performed. Two months later, the patient successfully underwent robot-assisted gastrectomy under general anesthesia with fentanyl, remifentanil, rocuronium, propofol and sevoflurane and showed no allergic reactions.

## Discussion

Anaphylaxis caused by benzodiazepines is very rare. In a recent report by the 6th National Audit Project (NAP6) in the UK [14], of the 199 agents identified as causative for perioperative anaphylaxis, the most common agents were antibiotics (94/199), neuromuscular blocking agents (65/199), and chlorhexidine (18/199), with no reports for benzodiazepines. On the other hand, there have been several reports of anaphylaxis caused by remimazolam, a new anesthetic, in recent years; 11 cases have been reported to date. The frequency of anaphylaxis caused by remimazolam is estimated to be 0.18% based on the report by Kim et al. [12]. The characteristics of previously reported remimazolam anaphylaxis cases and the present case are presented in Table 1. All patients had circulatory collapse, and cardiopulmonary resuscitation was needed in three patients, including our patient [9, 12]. Respiratory manifestations were observed in 58% (7/12) of patients, but bronchospasm has been reported in only one patient other than ours [8]; laryngeal edema was reported in 2 patients [10, 11]. According to the report by NAP6, hypotension was observed in all of the patients with perioperative anaphylaxis reported nationwide, bronchospasm/high airway pressure was reported in 48% of patients, and a reduced/absent capnography trace was reported in 33% of patients [14]. Based on these data, circulatory collapse was consistently observed in all cases of remimazolam anaphylaxis; however, bronchospasm due to remimazolam anaphylaxis was less frequent than other manifestations of anaphylaxis. Onset of anaphylaxis occurred within 5 min after remimazolam administration in 10 of the 12 cases. All patients were successfully treated with adrenaline. No biphasic anaphylaxis was reported.

**Table 1:** Characteristics of remimazolam anaphylaxis cases reported in the literature

**Authors**	**Age(years**	**Sex**	**Time to manifestation onset**	**Method of remimazolam administration**	**Total dose of remimazolam**	**Initial manifestations**	**Manifestations at any time during anaphylaxis**						**Elevated tryptase levels**	**Prior sensitization to remimazolam or midazolam**	**Skin test**
							**Cardiac arrest**	**Cardiovascular manifestations**	R**espiratory manifestations**	**Laryngeal edema**d	**Bronchospasm**	**Cutaneous manifestations**			
Uchida S et al. [7]	74	Male	5 minutes	4 mg intravenous (IV) infusion	4 mg	Circulatory collapse and oxygen desaturation	No	Yes	Yes	N/A	N/A	N/A	Yes	Remimazolam	Untested
Uchida S et al. [7]	59	Male	5 minutes	9 mg IV infusion in 3 divided doses	9 mg	Discomfort	No	Yes	No	N/A	N/A	No	Yes	No	Negative
Yamaoka M et al. [8]	78	Male	Immediately after induction of anesthesia	Continuous IV infusion at 12 mg/kg/h followed by at 1 mg/kg/h	N/A	Circulatory collapse, oxygen desaturation and high airway pressure	No	Yes	Yes	No	Yes	N/A	Yes	N/A	Positive
Hasushita Y et al. [9]	72	Male	15-20 minutes (6 minutes after tracheal intubation)	12 mg IV infusion followed by continuous IV infusion at 600 mg/h	N/A	Circulatory collapse and skin erythema	Yes	Yes	No	N/A	N/A	Yes	Yes	Midazolam	Positive
Hu X et al. [10]	41	Male	Within 1 minute	10 mg IV infusion	10 mg	Laryngeal stridor; erythema of the face, neck and chest; periorbital edema and lip swelling	No	Yes	Yes	Yes	N/A	Yes	Untested	N/A	Negative
Tsurumi K et al. [11]	32	Male	2 minutes	Continuous IV infusion at 6 mg/kg/h	12 mg	Facial flushing, circulatory collapse and oxygen desaturation	No	Yes	Yes	Yes	N/A	Yes	No	Midazolam	Positive
Kim KM et al. [12]	65	Male	5 minutes (2-3 minutes after tracheal intubation)	Continuous IV infusion at 12 mg/kg/h	97.5 mg	Circulatory collapse and ST-segment elevation	No	Yes	No	N/A	No	No	Yes	N/A	Negative
Kim KM et al. [12]	69	Male	5 minutes (2-3 minutes after tracheal intubation)	Continuous IV infusion at 12 mg/kg/h	76.8 mg	Circulatory collapse and ST-segment elevation	No	Yes	No	N/A	No	No	Yes	N/A	Untested
Kim KM et al. [12]	66	Male	6 minutes (2-3 minutes after tracheal intubation)	Continuous IV infusion at 12 mg/kg/h	56.3 mg	Circulatory collapse	Yes	Yes	No	N/A	No	No	Yes	N/A	Negative
Kim KM et al. [12]	23	Female	2-3 minutes	Continuous IV infusion at 12 mg/kg/h	25.6 mg	Facial flushing, skin rash, cough and chest tightnes	No	Yes	Yes	N/A	N/A	Yes	Untested	N/A	Negative
Kim KM et al. [12]	33	Female	2-3 minutes	Continuous IV infusion at 2 mg/kg/h	8.3 mg	Facial flushing, skin rash and dyspnea	No	Yes	Yes	N/A	N/A	Yes	Yes	N/A	Negative
Present case	75	Male	3-4 minutes	Continuous IV infusion at 6 mg/kg/h	8 mg	Bronchospasm/high airway pressure	Yes	Yes	Yes	N/A	Yes	Yes	Yes	Midazolam	Untested

Clinical information was collected and summarized from the text and figures in the references

*N/A* not available, *IV* intravenous

In the present case, anaphylaxis was characterized by severe bronchospasm/high airway pressure preceding circulatory collapse. If bronchospasm with remimazolam anaphylaxis develops, it may overlap the timing of tracheal intubation due to its rapid onset. Therefore, the diagnosis of remimazolam-induced anaphylaxis is sometimes challenging, as it can be confused with intubation complications. In addition, because remimazolam is a drug that can be administered continuously, a delay in responding to anaphylaxis can lead to more severe complications. Kim et al. reported a higher incidence of anaphylaxis in patients with a higher infusion rate [12].

Regarding circulatory management, The Australian and New Zealand College of Anaesthetists (ANZCA) and the Australian and New Zealand Anaesthetic Allergy Group (ANZAAG) recommend intravenous adrenaline under close monitoring for intraoperative anaphylaxis [15]. The dosages are 0.01–0.2 mg intravenously or 0.5 mg intramuscularly according to severity. If cardiac arrest occurs, 1 mg of adrenaline should be administered intravenously. In this case, we administered 0.5 mg of adrenaline intravenously, a dose lower than the recommendation. Although the patient was quickly resuscitated, we had difficulty regulating post-resuscitation hypertension; his systolic arterial blood pressure temporarily reached 300 mmHg and exceeded 250 mmHg for an extended period. In the other two previously reported cases of cardiac arrest due to remimazolam anaphylaxis [9, 12], both patients recovered spontaneous circulation with the administration of 1 mg of intravenous adrenaline, and post-resuscitation hypertension was greater than 200 mmHg. Therefore, in the case of cardiac arrest due to remimazolam anaphylaxis, a lower dose of intravenous adrenaline may be sufficient, and clinicians must be aware of the possibility of post-resuscitation hypertension.

It is essential to ascertain the allergy history of patients to prevent anaphylaxis. Anesthesiologists should fully review and evaluate past anesthesia records and allergy details [16]. If drugs suspected of inducing anaphylaxis are present, the best method of prevention is to avoid exposing patients to these drugs, including any cross-antigenic material [17]. Reports have suggested the presence of cross-reactivity between remimazolam and midazolam [9, 11]. In the present case, the patient had previously developed a severe allergic reaction thought to be caused by fresh-frozen plasma transfusion; however, midazolam had also been administered shortly before. Severe hypotension also occurred during the induction of anesthesia for cardiac surgery in which midazolam was administered. Considering the series of anaphylactic events, it is probable that the cause of the allergic reaction was midazolam, and the exposure to midazolam may have been the root cause of remimazolam anaphylaxis through the activation of an IgE-mediated pathway. However, we did not perform a skin prick test or intradermal test for midazolam and remimazolam; therefore, this assumption is not confirmed. On the other hand, the patient had a history of taking brotizolam, a benzodiazepine, but was not allergic to it. Regarding cross-reactivity among benzodiazepines, some reports showed cross-reactivity among benzodiazepines [18, 19], while others did not [20]. To date, cross-reactivity among benzodiazepines is poorly understood. The structures of remimazolam, midazolam and brotizolam are similar. However, remimazolam and midazolam are formed by a diazepine ring bonded to a benzene ring, whereas brotizolam is formed by a diazepine ring bonded to a thiophene ring; this structural difference may have caused a difference in cross-reactivity.

Some patients who experienced anaphylaxis induced by remimazolam had no history of exposure to remimazolam or midazolam [7] or negative skin test results for remimazolam [7, 10, 12]. Remimazolam preparations used worldwide, such as Anelem®, ByFavo®, and Aptimyda™, contain dextran 40 as an additive. Dextran occasionally causes an anaphylaxis-like, acute hypersensitivity reaction due to complement activation via immune complexes of non-IgE antibodies; this reaction is indistinguishable from IgE-mediated anaphylaxis in clinical findings [21]. It is possible that remimazolam anaphylaxis involves anaphylaxis via the IgE-mediated pathway and anaphylaxis-like reactions caused by dextran via the non-IgE-mediated pathway.

A limitation of our case report is that we did not perform either skin prick tests and intradermal tests for remimazolam and midazolam due to considerations of patient safety. Then we also did not perform intradermal tests for any of the suspected drugs including latex which is also a common cause of anaphylaxis. Therefore, the causative agent was not confirmed by allergic tests. However, the other suspected drugs yielded negative results on the prick tests, and those drugs could be used clinically during anesthetic management of the patient during subsequent surgery without causing an allergic reaction. Given the patient’s history, there is a very high likelihood that the cause of anaphylaxis is remimazolam. However, the gold standard for post-anaphylaxis management is to make an accurate diagnosis and to provide patients with detailed information about their anaphylaxis. Our review shows that the positive rate of skin tests for remimazolam is low (Table 1), and according to a recent report, even skin tests have a positive rate of only about 57% for diagnosing intraoperative anaphylaxis. Therefore, it is necessary to combine the skin test and various tests (e.g., specific antibody test and basophil activation test) to improve the diagnostic accuracy [22].

## Conclusion

We report a case and literature review of remimazolam-induced anaphylaxis. Remimazolam is an ultrashort-acting sedative; however, it can cause life-threatening anaphylaxis. In addition, its cross-reactivity with other benzodiazepines is not known. To increase the safety of this drug, further research and more experience in its use are needed.

### Supplementary Information

Additional file 1. Video of bronchoscopy during anaphylaxis.

Additional file 2. Video of bronchoscopy before extubation in the intensive care unit.

Additional file 3. Skin prick test results.

Additional file 4. List of agents used during this patient’s perioperative periods.

## Data Availability

On reasonable request, data regarding this patient can be obtained from the corresponding author.
