# Supplementary material for: Remimazolam anaphylaxis in a patient not allergic to brotizolam: a case report and literature review
Source: BMC Anesthesiol. 2024 Jun 8;24:204. doi: 10.1186/s12871-024-02591-w (PMC11161983; doi:10.1186/s12871-024-02591-w)
Supplement: Supplementary file 3 — Additional file 3. Skin prick test results. [file 12871_2024_2591_MOESM3_ESM.pdf]

Supplementary Information

Additional File 3.

Skin prick test results.

Drugs	Drug concentration (mg/ml)	Diameter of flare (mm)	Diameter of wheal (mm)	Interpretation
Negative control (saline)		1	0	(-)
Positive control (histamine)		15	7	(+)
Rocuronium	0.1/1/10	1/2/2	0/0/0	(-)
Remifentanil	0.001/0.01/0.1	1/1/2	0/0/0	(-)
Propofol	10	1	0	(-)
Cefazolin	10	2	0	(-)
Suxamethonium	0.1/1/10	2/2/2	0/0/0	(-)
Lidocaine	10	2	0	(-)
Bupivacaine	5	2	0	(-)
Popscaine	2.5	2	0	(-)
