# Supplementary material for: Remimazolam anaphylaxis in a patient not allergic to brotizolam: a case report and literature review
Source: BMC Anesthesiol. 2024 Jun 8;24:204. doi: 10.1186/s12871-024-02591-w (PMC11161983; doi:10.1186/s12871-024-02591-w)
Supplement: Supplementary file 4 — Additional file 4. List of agents used during this patient’s perioperative periods. [file 12871_2024_2591_MOESM4_ESM.pdf]

Supplementary Information

Additional File 4.

List of agents used during this patient's perioperative periods.

**Clinical course**	**Induction of anesthesia for cardiac surgery**	**Maintenance of anesthesia during cardiac surgery**	**On admission to ICU after cardiac surgery**	**During ICU stay after anaphylaxis**	**Induction of anesthesia for gastric surgery**	**Maintenance of anesthesia during gastric surgery after anaphylaxis**	**All causative agents**
**Possibility of anaphylaxis**	±	-	+	-	+	-	
**Anaphylactic signs**	Severe drop in blood pressure during induction of anesthesia		Rash on both upper extremities, severe drop in blood pressure, and elevated airway resistance		Rash on abdomen and thighs, severe drop in blood pressure, and elevated airway resistance		
**Agent list in the order of administration during events**					Remimazolam		Remimazolam
	Midazolam		Midazolam				Midazolam
	Fentanyl	Fentanyl		Fentanyl	Fentanyl	Fentanyl	Fentanyl
	Remifentanil	Remifentanil			Remifentanil	Remifentanil	Remifentanil
	Rocuronium	Rocuronium		Rocuronium	Rocuronium		Rocuronium
				Propofol		Propofol	Propofol
				Dexmedetomidine			Dexmedetomidine
			FFP				FFP

ICU, intensive care unit; FFP, fresh-frozen plasma.

Agents in the striped area were administered during periods when the patient did not experience anaphylactic reactions and were considered safe.
